# Primary squamous cell carcinoma of the stomach: A case report

**DOI:** 10.3892/ol.2014.2492

**Published:** 2014-09-01

**Authors:** SUN HWI HWANG, JUNG HEE LEE, KYUNGBIN KIM, DONG HUN SHIN, JEE YEON KIM, MEE YOUNG SOL, KYUNG UN CHOI

**Affiliations:** 1 Department of Surgery, Pusan National University Yangsan Hospital, Yangsan, Gyeongsangnam-do 626-770, Republic of Korea; 2 Department of Pathology, Pusan National University Yangsan Hospital, Yangsan, Gyeongsangnam-do 626-770, Republic of Korea; 3 Department of Pathology, School of Medicine, Pusan National University, Yangsan, Gyeongsangnam-do 626-870, Republic of Korea

**Keywords:** squamous cell carcinoma, stomach

## Abstract

Pure squamous cell carcinoma (SCC) of the stomach is rare and resembles SCC arising elsewhere in the body. The pathogenesis of SCC remains unclear and controversial. At present, <100 cases of primary SCC of the stomach have been reported. The current study presents a case of SCC of the stomach in a 61-year-old male. Total gastrectomy was performed and a 7.0×6.7×4.5-cm tumor with a superiorly located ulcer was identified in the cardia. Upon histological examination, a moderately-differentiated SCC was observed. Tumor cells extended to the serosa, and the perigastric regional lymph node was also involved. No evidence of human papillomavirus (HPV) or Epstein-Barr virus (EBV) infection was identified using a DNA microarray and *in situ* hybridization, respectively. A post-operative computed tomography scan four months after the gastrectomy revealed tumor recurrence and dissemination of the tumor to the jejunum and pancreas. The patient succumbed to the disease six months later despite the administration of low-dose adjuvant 5-fluorouracil/cisplatin chemotherapy.

## Introduction

The incidence of primary squamous cell carcinoma (SCC) of the stomach ranges between 0.04 and 0.09%, making the disease an extremely rare entity (1). SCC was first identified in 1895, and <100 cases have been reported worldwide (2–15). Although theories exist regarding its development in the stomach (2,6,7), the pathogenesis of SCC remains unclear. Primary SCC of the stomach is often diagnosed at a late stage, and its prognosis is generally poor. Furthermore, no effective adjuvant chemotherapy has been identified.

## Case report

**Figure 1:**
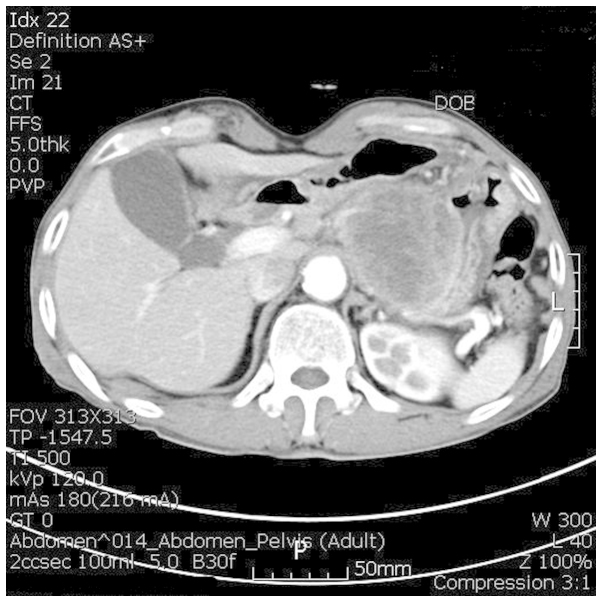
Computed tomography revealing a heterogeneously-enhanced lesion of the cardia.

**Figure 2:**
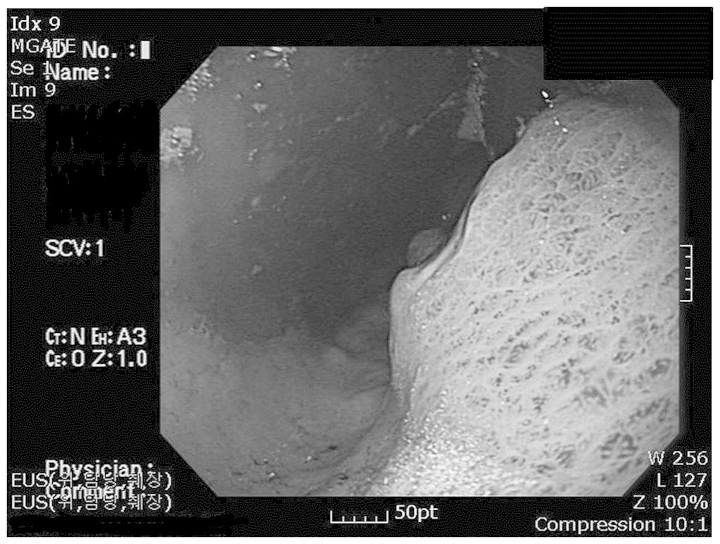
Endoscopic observation revealing a large tumor located predominantly in the cardia, which did not involve the gastroesophageal junction.

A 61-year-old male was admitted to the Pusan National University Yangsan Hospital (Yangsan, Korea) with a six-month history of weight loss, totaling ~6 kg. The patient exhibited no symptoms, including abdominal pain, nausea, vomiting or melena. Furthermore, a physical examination and routine laboratory tests on admission revealed no specific abnormalities. The patient underwent an abdominal computed tomography (CT) scan, which demonstrated a heterogeneously-enhanced tumor mass of 6.5 cm (Fig. 1). Endoscopic examination and endoscopic ultrasonography of the gastrointestinal tract revealed a submucosal tumor mass of 6 cm, with surface ulceration on the fundus and cardia of the stomach (Fig. 2). These observations indicated that the tumor was derived from non-epithelial cells, such as those that derive a gastrointestinal stroma tumor or malignant lymphoma. Notably, a biopsy specimen revealed the diagnosis of SCC.

**Figure 3:**
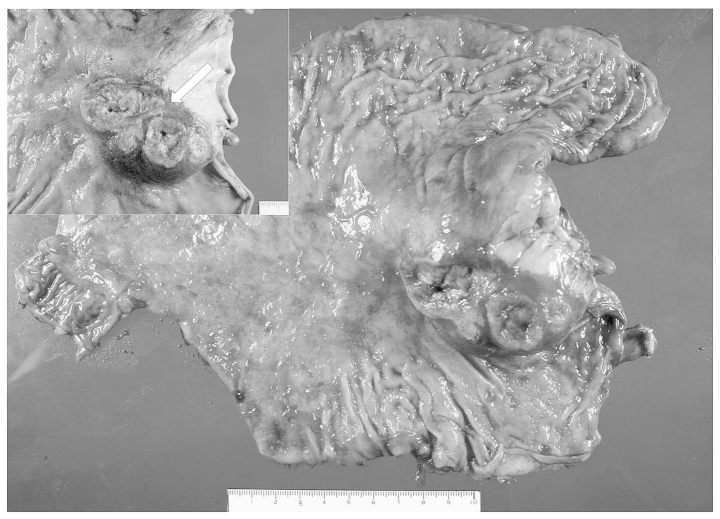
Gross findings of the resected stomach. A definite band of normal gastric mucosa was identified between the gastroesophageal junction and the carcinoma (shown by the arrow on the inset image).

**Figure 4:**
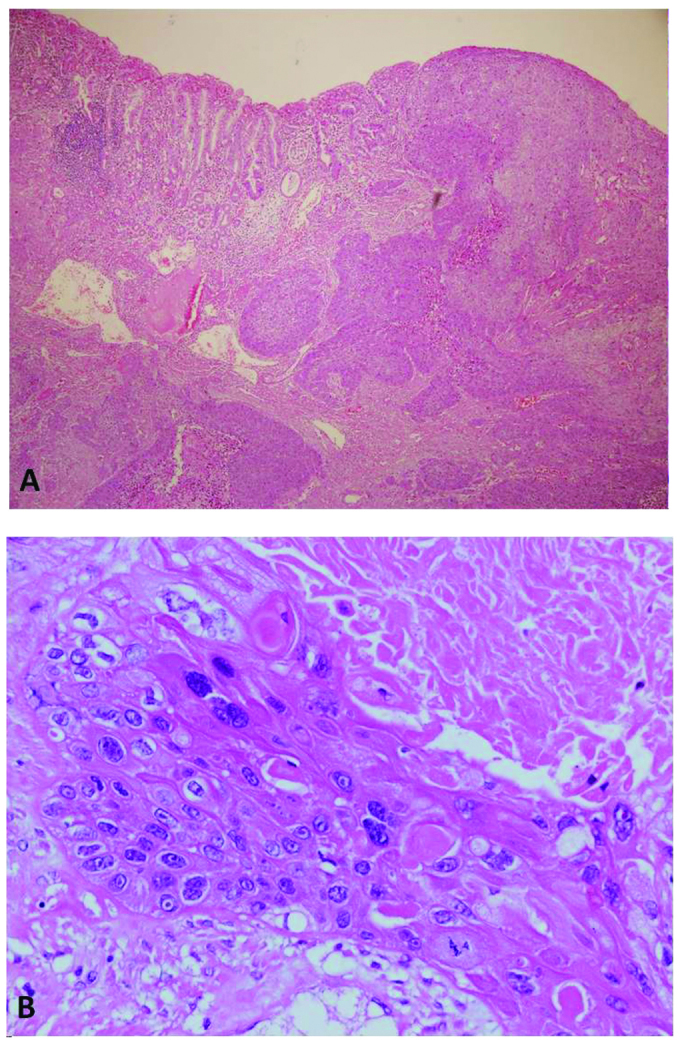
Microscopic observations of the tumor. (A) The tumor exhibited infiltrating tumor nests and overlying SCC *in situ* (stain, hematoxylin and eosin; magnification, ×20) and consisted of (B) moderately-differentiated SCC, exhibiting a keratin formation and intercellular bridges (stain, hematoxylin and eosin; magnification, ×400). SCC, squamous cell carcinoma.

The patient underwent a total gastrectomy. The resected stomach exhibited a 7.0×6.7×4.5-cm tumor in the cardia, in the vicinity of the gastroesophageal junction (GEJ). The tumor exhibited a superiorly located ulcer and infiltrated the stomach wall (Fig. 3). A 0.7-cm tumor-free section was identified between the GEJ and the tumor. Histologically, the tumor was composed of moderately-differentiated squamous cells with keratinization and intercellular bridges in certain sections (Fig. 4). No evidence of glandular differentiation was identified within the tumor. SCC *in situ* without squamous metaplasia was observed adjacent to the tumor. The tumor was not contiguous with the squamous mucosa of the distal esophagus and was growing predominantly in the submucosal and muscle layers. The serosal layer exposed to the tumor and the gastric mucosa exhibited focal invasion by the tumor. One section of the lymph nodes of the lesser curvature exhibited metastatic SCC.

To investigate the role of human papillomavirus (HPV) and Epstein-Barr virus (EBV) infection in the carcinogenesis of SCC arising in the stomach, DNA microarray and *in situ* hybridization, respectively, were used for detection. However, no evidence of HPV or EBV infection was identified in the patient.

One month after the surgery, combination chemotherapy consisting of cisplatin (90 mg every 15 min) and 5-flourouracil (1,500 mg every 10 h) was administered for five days every 21 days. The patient received two cycles of chemotherapy. A follow-up abdominal CT scan four months later revealed tumor invasion of the jejunum and pancreas, enlargement of abdominal lymph nodes and multiple foci of liver metastasis. The patient succumbed to the disease six months later.

## Discussion

Primary SCC of the stomach consists of only SCC as opposed to one consisting of adenocarcinoma, the most common malignancy in the stomach. The clinicopathological features of 90 patients with SCC are shown in Table I. Data were selected from previous studies in the English literature (2–15). The incidence of SCC was three times higher in the male patients compared with the females. The mean age of the patients was 59.7 years and the tumor size ranged between 2.1 and 17 cm (mean size, 7.1 cm).

In SCC proximal to the GEJ, evidence of its gastric origin must be present. The Japanese Gastric Cancer Association proposed the following criteria for the diagnosis of primary SCC (16): The tumor must consist of only SCC without adenocarcinoma, and any tumor close to the GEJ must not be diagnosed as gastric SCC without evidence that it originated in the stomach. The diagnosis of primary gastric SCC requires that normal gastric mucosa is present between the GEJ and SCC of the stomach. The present case matched these criteria and thus was diagnosed as primary SCC of the stomach cardia. However, Parks (2) proposed that SCC occurring in the cardia must not be considered as a primary gastric carcinoma, as it may arise from the distal esophagus or misplaced islands of squamous cells in the cardia. A number of previous studies have proposed that it may be referred to as gastric SCC when the lesion is completely separate from the esophageal epithelium (3–5).

A number of theories regarding the origin of SCC of the stomach have been proposed, including totipotent stem cells, squamous metaplasia, foci of heterotopic squamous epithelium and the overgrowth of a squamous epithelium element in a primary adenocarcinoma. However, the pathogenesis of SCC remains unclear. We favor the theory that SCC originates from the squamous metaplastic epithelium or heterotopic squamous epithelium, as the presence of squamous epithelium in the cardia has been reported (6), squamous metaplasia has been observed with SCC (7) and evidence of SCC *in situ* was identified during pathological examination in the present case.

Takita *et al* (17) proposed that EBV infection may be involved in the pathogenesis of certain cases of gastric SCC. In this study, a liquid hybridization assay for HPV infection and a polymerase chain reaction for EBV infection was performed and revealed the presence of EBV infection in surgical specimens of the tumor. However, no evidence of HPV or EBV infection was identified in the present case when using DNA microarray for HPV infection and *in situ* hybridization for EBV infection.

A standard chemotherapy regimen for this disease has not yet been established, and only one previous study has demonstrated the efficacy of chemotherapy against the tumor (4). The prognosis of primary SCC of the stomach has not been clearly defined. However, previous studies have indicated a poor prognosis for this disease, as the majority of lesions are detected at an advanced stage, with marked infiltrative growth. The present case exhibited disease progression despite chemotherapy administration.

## Figures and Tables

**Table I:** Characteristics of patients with primary squamous cell carcinoma of the stomach (n=90).

Parameter	Value
Mean patient age, years	59.7
Gender, n (%)	
Male	63 (70.0)
Female	18 (20.0)
Not stated	9 (10.0)
Tumor location, n (%)	
Proximal	22 (24.4)
Middle	22 (24.4)
Distal	18 (20.0)
Not stated	28 (31.1)
Mean tumor size, cm	7.1
